# Heritability of longevity in Large White and Landrace sows using continuous time and grouped data models

**DOI:** 10.1186/1297-9686-42-13

**Published:** 2010-05-13

**Authors:** Gábor Mészáros, Judit Pálos, Vincent Ducrocq, Johann Sölkner

**Affiliations:** 1 Division of Livestock Sciences, University of Natural Resources and Applied Life Sciences, Gregor Mendel Str.33, 1180, Vienna, Austria; 2 UMR 1313 INRA, Génétique Animale et Biologie Intégrative, 78352 Jouy-en-Josas, France

## Abstract

**Background:**

Using conventional measurements of lifetime, it is not possible to differentiate between productive and non-productive days during a sow's lifetime and this can lead to estimated breeding values favoring less productive animals. By rescaling the time axis from continuous to several discrete classes, grouped survival data (discrete survival time) models can be used instead.

**Methods:**

The productive life length of 12319 Large White and 9833 Landrace sows was analyzed with continuous scale and grouped data models. Random effect of herd*year, fixed effects of interaction between parity and relative number of piglets, age at first farrowing and annual herd size change were included in the analysis. The genetic component was estimated from sire, sire-maternal grandsire, sire-dam, sire-maternal grandsire and animal models, and the heritabilities computed for each model type in both breeds.

**Results:**

If age at first farrowing was under 43 weeks or above 60 weeks, the risk of culling sows increased. An interaction between parity and relative litter size was observed, expressed by limited culling during first parity and severe risk increase of culling sows having small litters later in life. In the Landrace breed, heritabilities ranged between 0.05 and 0.08 (s.e. 0.014-0.020) for the continuous and between 0.07 and 0.11 (s.e. 0.016-0.023) for the grouped data models, and in the Large White breed, they ranged between 0.08 and 0.14 (s.e. 0.012-0.026) for the continuous and between 0.08 and 0.13 (s.e. 0.012-0.025) for the grouped data models.

**Conclusions:**

Heritabilities for length of productive life were similar with continuous time and grouped data models in both breeds. Based on these results and because grouped data models better reflect the economical needs in meat animals, we conclude that grouped data models are more appropriate in pig.

## Background

Length of productive life is important from economical, herd-health and animal welfare points of view in sustainable animal production. Intensive selection on production and reproduction traits without considering functional and exterior traits can lead to decreased longevity [1,2]. In Austria, exterior traits are taken in account during selection of replacement gilts before the first insemination. At this stage only a negative selection is carried out, without any official recording for later use. However, data on length of productive life and number of piglets born/weaned are routinely collected and available for Herdbook sows. The total number of piglets born or weaned can also be used to express the lifetime production of sows, but genetic evaluation of litter size is already implemented in the Austrian system.

Length of productive life measured as the number of days between first farrowing and culling has been analyzed in several studies using either Cox [3] or Weibull models [4]. During the productive life of sows, the period between weaning and conception can be non-productive and optimally, it should be kept as short as to ensure the highest number of litters. Using conventional measurements of lifetime (i.e. number of days between first farrowing and culling), it is not possible to differentiate between productive and non-productive days during a sow's lifetime, which can lead to less pertinent results in breeding value estimation [5]. For this reason, a sow's productive life would be better expressed as the number of completed parities.

This approach requires rescaling of the time axis from a continuous scale into several discrete classes. The consequence of this approach is that Cox and Weibull models will no longer be valid, because these usual approaches assume continuity of the baseline hazard distribution and/or absence of ties between ordered failure times [6]. Instead, grouped survival data (discrete survival time) models introduced by Prentice and Gloeckler [7] can be used. Grouped data models have been used in beef cattle [8], rabbits [9] and dairy cattle to evaluate fertility traits [10], but not for length of productive life in pigs.

The aim of this study is to compare the performance of Weibull and grouped data models and to estimate heritabilities using different genetic models for Large White and Landrace sows.

## Methods

### Data

**Figure 1:**
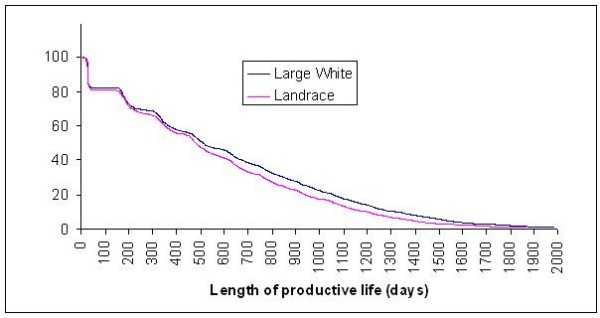
**Survival in percents for Large White and Landrace populations**.

Length of productive life was analyzed for 12319 Large White sows originating from 838 boars and 4348 dams and for 9833 Landrace sows originating from 457 boars and 2236 dams. Overall survival for both populations is shown in Figure 1. All sows were purebred and were part of the herd book in nucleus or multiplier herds. Records from breeding farms represented 10% of the Landrace sows and 40% of the Large White sows. Landrace and Large White animals are used on breeding farms in Lower and Upper Austria, crossed with Large White animals on the multiplier level. In Steiermark, the Large White breed is used on both breeder and multiplier levels. In some cases, breeding farms also produce F1 sows if these can be marketed for a good price. For these reasons, breeding and multiplier farms were difficult to distinguish and thus, in this study, they were analyzed jointly. In all cases, F1 sows are mated with Pietrain boars on the piglet producer level in Austria.

For the Weibull model, length of productive life was defined as the number of days between first farrowing and culling. We assumed that culling took place either at the last weaning of the sow or 28 days after the last farrowing if the number of weaned piglets was known, but if both weaning date and number of weaned piglets were missing, culling date was set one day after the last farrowing. The choice of setting the culling date at 28 days after the last farrowing for sows with incomplete weaning date was based on the average nursing period in the whole dataset.

Two intervals per parity were used for the grouped data model: from farrowing to weaning and from weaning to the next farrowing. Intervals were numbered sequentially from 1 up to 18 (i.e. sow alive after the 9^th ^weaning). Animals were censored either at the date of the last weaning, if they were alive at the time of data collection or at the 9^th ^weaning if they were alive at the 10^th ^farrowing. Only sows born after 1995 were included in the evaluation and age at first farrowing between 250 and 550 days was required.

The analysis was carried out using a proportional hazards model (assumed to be either the Weibull model or a grouped data model) with the Survival Kit v6 program package [11].

### Continuous time model

The continuous length of productive life was analyzed with the following Weibull model:

where h*y_*i *_is the time-dependent random effect of herd and year of farrowing assumed to follow a log-gamma distribution, *aff*_*j *_the fixed time independent effect of class of age at first farrowing, par*pigl_*k *_the time-dependent effect of interaction between parity and relative number of piglets (see below for detailed description), *hs*_*l *_the time-dependent effect of annual herd size change. The random genetic component *g*_*m *_differed, defining an animal, sire, sire-maternal grandsire, sire-dam or sire-maternal grandsire-dam within a maternal grandsire genetic model.

For age at first farrowing, 33 classes were created with one-week intervals, where the first group contained animals up to 43 weeks of age and the last group contained animals older than 75 weeks at first farrowing.

Parity and classes for piglets born alive relative to the annual herd's mean were combined into an interaction term and included into the model (similar to [12]). This was done in several steps:

Step 1: The number of piglets born alive was corrected for the first farrowing litter size. This was necessary, because the average number of piglets born at first farrowing is lower than that at later farrowing;

where n is the number of the parity ranging between 2 and 6. Parities 6 and higher were treated in the same way, because of very similar coefficients. The values for the coefficients between parity 2 and parity 6 were: 1.055, 1.0877, 1.0922, 1.0853 and 1.0473;

where *cnp *is the corrected number of piglets and *n *is the number of the parity ranging between 2 and 6.

Step 2: The average number of piglets for each year within each herd (h × y) was computed;

Step 3: The previously corrected numbers of piglets born alive (in Step 1) were compared to the annual herd's mean (computed in step 2).

where *cnp *is the corrected number of piglets born alive and *m *is the number of the parity ranging between 1 and 9 (maximal parity after censoring).

Ten classes (relative piglet classes or RPC) were created according to percentage deviation from the herd mean, as follows: <75%, 75-85%, 85-90%, 90-95%, 95-100%, 100-105%, 105-110%, 110-115%, 115-125% and >125%. RPC were inserted in the model as an interaction term with the parity number. Classes were recoded as numbers with three digits, where the first digit denoted the parity number (from 1 to 9), and the last two digits the RPC class (from 1 to 10).

Similarly the annual herd size changes were grouped into eight classes, according to number of farrowing per herd and year, where January 1^st ^of each year was considered as cut point. In case the number of farrowings was equal or below 10, no change was accounted for that particular year. The bounds for classes were: decrease by more than 50%, decrease by 30-50%, 30-10%, between decrease by 10% and increase by 10%, increase by 10-30%, 30-50%, 50-100% and increase by 100% and more.

Longevity of sows can also be influenced by index values on growth traits but since these indexes are not routinely saved in Austria, we could not include them into the models.

### Grouped data model

Grouped data models are a special case of proportional hazards models, where failure times are grouped into intervals A_i _= [a_i-1_, a_i_), i = 1, ..., r with a_0 _= 0, a_r _= +infinite and failure times in A_i _are recoded as t_i_. Therefore the regression vector is assumed to be time-dependent but fixed within each time interval [7].

For the grouped data models, the same effects as for the Weibull model were used.

### Genetic models and heritability computation

For both Large White and Landrace databases, the same structure of fixed and random effects was used. All models accounted for pedigree information up to the third generation of ancestors. The genetic variance was estimated as the mode of its approximate posterior density after Laplace integration of the other parameters [13]. At the same time, the mean, variance and skewness of this posterior density were obtained. Knowing these three parameters, makes it possible to draw the posterior density of the variance component using a Gram-Charlier approximation.

The standard deviation of the posterior density can be interpreted as a conservative estimate of the standard error. From this, the standard error of the heritabilities was computed using the Delta method (see e.g. [14]).

### Sire model

In this case, the sow's sire was included in the model, accounting for 1/4 of the genetic variance. To be correct, the model implicitly assumes that mates are non-related, non-inbred, non-selected and that each dam has one recorded progeny only. The pedigree file contained the sires' sire and sires' maternal grandsire.

The effective heritability was computed from the sire's variance as in Yazdi et al. [15]. The effective heritability accounts for censoring in contrast with the equivalent heritability which conceptually assumes that all animals have died. The effective reliability is useful to compute expected reliabilities of EBV as a function of the expected number of animals still alive at a given time. The effect of the herd-year was treated as a time-dependent random variable assuming a loggamma distribution in all cases. The following equation was used:

where  is the genetic variance, var(**h **× **y**) the herd year variance, *p *the proportion of uncensored animals and genetic variance = 4 * sire variance.

### Sire - maternal grandsire model

This was similar to the sire model, but the sow's maternal grandsire was also included in the model and recoded jointly with the sires. This model accounts for 1/4 + 1/16 = 5/16 of the genetic variance under the same assumptions as the sire model (i.e. mates are non-related, non-inbred, non-selected and each dam has one progeny only). Additionally dams can be related and selected through their sire (i.e. the maternal grandsire of the progeny).

The pedigree file had the same structure and the heritability was computed with the equation:

where  is the genetic variance, var(**h **× **y**) the herd year variance, *p *the proportion of uncensored animals and genetic variance = 4 * sire variance. The additional 1/16 genetic variance in the denominator stands for the maternal grandsire's variance.

### Sire - dam model

Here both the sire and dam were included in the model, but recoded together in the data preparation step. Both sire and dam account for half of the genetic variance, and full-sibs are therefore recognized as being more similar than half-sibs. For both parents of the sow, their sire and dam were included in the pedigree file. The effective heritability was computed as:

where  is the genetic variance, var(**h **× **y**) the herd year variance, *p *the proportion of uncensored animals and genetic variance = 4 * sire variance. The genetic variance was multiplied by 2 in the denominator (compared to the sire model) because the sire and dam variances are assumed equal.

### Sire - maternal grandsire - dam within maternal grandsire

This model is in some sense a compromise between sire-maternal grandsire and sire-dam models, because the relationship matrix involves only males and it still accounts for repeated records. The sow's sire and maternal grandsire are recoded together in the data preparation step. In the final model the sire, maternal grandsire and dam of the sow are included as separate random effects.

This model does not account for the Mendelian sampling term of the animal but in contrast with the sire-maternal grandsire model, sisters can have different genetic values and more than one progeny each in which case a non genetic maternal effect is also accounted for. The main difference with a sire-dam model including a maternal effect is that dams are considered as related only through their sire (i.e. maternal grand dams are supposed to be unselected and to have only one progeny each).

The heritability was computed as:

where  is the genetic variance, var(**h **× **y**) the herd year variance, *p *the proportion of uncensored animals,  the dam within maternal grandsire variance and genetic variance = 4 * sire variance. The additional 1/16 genetic variance in the denominator stands for the maternal grandsire variance.

### Animal model

In this case, the animal effect is responsible for the entire genetic variance and all its ancestors are accounted for. It is included in the evaluated model as a random effect, as well as the pedigree file together with its sire and dam. The heritability is computed as follows:

where  is the genetic variance, var(**h **× **y**) the herd year variance, *p *the proportion of uncensored animals.

## Results

### Results for fixed effects

A brief statistical overview of the databases is presented in Table 1. The total proportion of right censored sows was 26.4% in the Large White and 22.3% in the Landrace database. Landrace sows lived 92 days longer and completed 0.56 more parities on average, compared to Large White sows. Standard deviations were large in both cases. Large White sows had approximately 0.5 more piglets per farrowing. The average age at first farrowing was similar in both populations.

**Table 1:** Statistical overview

	Large White ^a^		Landrace ^b^	
	
	mean	std	mean	std
Parity	4.14	2.87	4.70	3.03
non-censored	4.31	2.94	4.81	3.08
censored	3.55	2.52	4.09	2.62

LPL at last litter (days)	503	456	595	481
non-censored	531	467	615	490
censored	401	397	485	412

Piglets born alive per parity	10.57	1.62	9.96	1.47
non-censored	10.35	1.53	9.87	1.45
censored	11.36	1.67	10.50	1.51

Age at first farrowing (days)	378	43	367	37
non-censored	378	44	367	37
censored	380	40	370	36

^a^total number of records: 12319, number of non-censored: 9645, number of censored: 2674; ^b^total number of records: 9833, number of non-censored: 8332, number of censored: 1501

**Figure 2:**
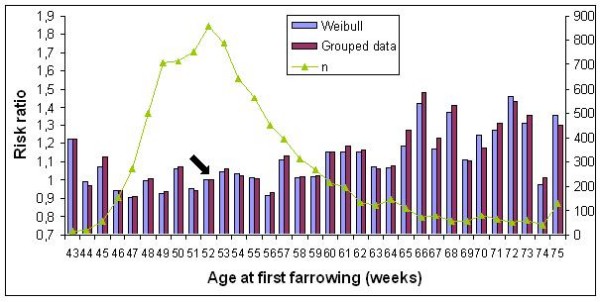
**Risk ratios for classes of age at first farrowing in Large White sows**. n = number of uncensored observations; the arrow indicates the reference class.

**Figure 3:**
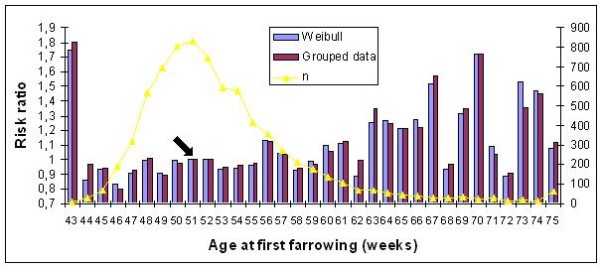
**Risk ratios for classes of age at first farrowing in Landrace sows**. n = number of uncensored observations; the arrow indicates the reference class.

Similar trends for the risk ratios of age at first farrowing were found for all models and also across breeds (Figures 2 and 3). A high risk of culling was observed for sows which had their first litter at a very young age, compared to the reference class (risk ratio = 1) at week 52 for Large White and week 51 for Landrace sows. After this, a longer period with a moderate risk follows, approximately till 59 weeks of age, after which the risk of culling increased again.

**Figure 4:**
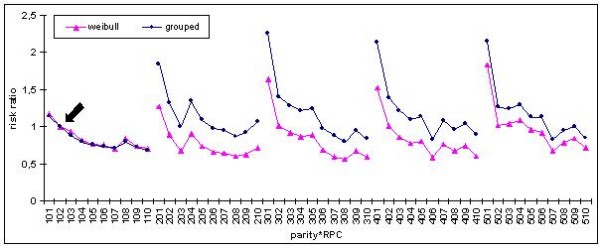
Risk ratios for parity*RPC in Large White. Note: The three digit classes on the x axis stand for parity (hundreds) and class of relative piglet classes (tens and ones); for example 201 is the lowest RPC class in parity 2; the arrow indicates the reference class.

As for the interaction between parity and RPC, the risk ratios for classes were different between breeds and model types (continuous time or grouped data). The risk ratios from the grouped data models were similar during the first parity, but were higher later on, compared to continuous time models, as showed in Figure 4. Within breed and model type, the risks of culling for the classes were similar, regardless of the genetic component.

Within parities the risk was highest for sows with a litter size below 75% of the herd's average in a given year, with only a slight decrease for sows with a higher number of piglets. First parities of both Large White and Landrace sows seemed to be exceptions from this pattern. In Large White sows, only a slight decrease of the risk ratio was observed throughout the classes and the peak value for the worst class was much lower. In Landrace sows, the risk ratio was much higher for sows with a litter size below that of the farm's average, but for the other classes no clear tendency was observed.

For Large White sows, the risk ratios were similar for farrowings 2 to 4 in both Weibull and grouped data models and from parity 6 (not shown) it increased. However, during the last parity the risk dropped for the grouped data model, compared to the Weibull model. For Landrace sows, the risk ratios were similar only for the first three parities in grouped data models, and increased from parity 4 onwards. Again during the last parity (not shown) the risk ratio dropped considerably. Unlike the other cases, the risk of culling decreased between parities for the Weibull model in the Landrace breed.

**Figure 5:**
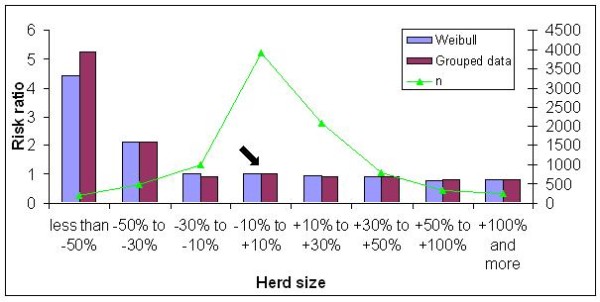
**Risk ratios for annual herd size change in Large White sows**. The arrow indicates the reference class.

The risk of culling in both populations was highest for sows from herds with a rapid size decrease (Figure 5), as expected. The opposite tendency was not clear for growing farms: the risk remained virtually the same whether the herd size decreased by less than 30% or increased to by whatever percent.

### Heritabilities

**Figure 6:**
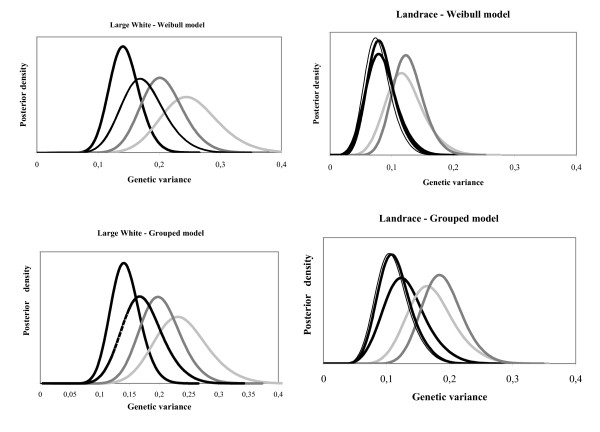
**Posterior density curves for Landrace and Large White sows**. wide black line: animal model; dashed line: sire-maternal grand sire model; thin black: thin black line: sire-maternal grand sire (dam within maternal grand sire mode; dark grey line: sire model; light grey line: sire-dam model.

The estimated genetic variances and heritabilities together with their standard errors for all models in both breeds are given in Tables 2 and 3. The posterior density of the genetic variance for each model is presented in Figure 6. These figures show that all genetic variances are statistically different from 0 and that the confidence intervals (credible sets) are quite wide. For a given breed and type of model (Weibull vs. grouped data), the posterior densities overlap to a large extent. In general, heritabilities differ slightly depending on the breed and genetic model used. For a given genetic model, genetic variances and heritabilities are extremely similar in grouped data and continuous time models for the Large White breed, but they are systematically higher in the grouped data model for the Landrace breed.

**Table 2:** Genetic variances and heritabilities for Large White (herd*year var = 0.325)

Genetic models	Continuous scale		Grouped data scale	
	
	variance (std deviation)	heritability (std deviation)	variance (std deviation)	heritability (std deviation)
Animal	0.140 (0.024)	0.077 (0.012)	0.140 (0.025)	0.077 (0.012)

Sire mgs dam	0.169 ^a ^(0.018)	0.097 (0.023)	0.166 ^b ^(0.017)	0.095 (0.023)

Sire dam	0.202 (0.035)	0.113 (0.018)	0.197 (0.034)	0.111 (0.018)

Sire mgs	0.171 (0.018)	0.098 (0.020)	0.167 (0.017)	0.097 (0.019)

Sire	0.246 (0.023)	0.141 (0.026)	0.230 (0.022)	0.132 (0.025)

^a^dam within maternal grandsire (mgs) variance = 0.016; ^b^dam within maternal grandsire variance = 0.015

**Table 3:** Genetic variances and heritabilities for Landrace (herd*year var = 0.233)

Genetic models	Continuous scale		Grouped data scale	
	
	Variance (std deviation)	Heritability (std deviation)	Variance (std deviation)	Heritability (std deviation)
Animal	0.078 (0.025)	0.049 (0.015)	0.122 (0.033)	0.074 (0.019)

Sire mgs dam	0.074 ^a ^(0.011)	0.047 (0.014)	0.104^b ^(0.013)	0.066 (0.016)

Sire dam	0.123 (0.025)	0.078 (0.015)	0.184 (0.032)	0.114 (0.019)

Sire mgs	0.078 (0.011)	0.050 (0.014)	0.108 (0.013)	0.069 (0.017)

Sire	0.115 (0.015)	0.074 (0.020)	0.163 (0.018)	0.105 (0.023)

^a^dam within maternal grandsire (mgs) variance = 0.027; ^b^dam within maternal grandsire variance = 0.029

For the Landrace breed, heritabilities range between 0.05 and 0.08 (s.e. 0.014 - 0.020) for the continuous time and between 0.07 and 0.11 (s.e. 0.016 - 0.023) for the grouped data models. Heritabilities for Large White sows range between 0.08 and 0.14 (s.e. 0.012 - 0.026) for the continuous time and between 0.08 and 0.13 (s.e. 0.012 - 0.025) for the grouped data models. Whatever the breed, heritabilities are highest for the sire and sire-dam models. The sire-mgs and the sire-mgs-dam within mgs models gave similar, but smaller heritabilities. For the latter model, the dam within mgs variance is supposed to include 3/16 of the genetic variance plus the maternal non genetic effect variance if such an effect exists. In fact, for the Large White breed, the estimated within mgs variance of the dam (0.016/0.015) was smaller than 3/16 of the estimated genetic variance (0.031 for both models). This inconsistency was not observed in the Landrace breed. Hence, if a maternal non genetic effect exists, it should be very small. Finally, the lowest genetic variances and heritabilities were obtained with the animal model.

## Discussion

The average length of productive life in the whole dataset was 503 days for Large White and 596 days for Landrace sows, with large standard deviations. Results for the Landrace breed were comparable, but for the Large White breed, they were lower than those reported in the literature i.e. 617 days in [16] and 602 days in [4]. Such results are heavily dependent on the amount of censored records and the length of the study period.

Prolonged productive life is important for two main reasons: 1. in general, the number of piglets born during farrowing 3 and 4 is higher than during the first farrowings, which means that with a higher proportion of older sows, piglet production increases. 2. On breeding farms with short generation intervals, it is especially important that only a low proportion of sows be culled for health and fertility problems, and the remaining ones be selected according to production traits, like litter size, fattening or carcass traits.

Our results show that age at first farrowing affects the risk of culling only for animals that have their first litter very early or late in life. A very young sow is not prepared to give birth because of its body development is not sufficient. This particular problem seems to affect only sows farrowed before 43 weeks of age. This result is in agreement with [17] in Danish Landrace herds for which in the case of an early first mating i.e. before 210 days of age, the risk for culling was higher than for sows mated later. After the 43rd week, the risk ratio dropped to a level around that of the reference class. Age at first farrowing increased the risk of culling again, for sows older than 60 weeks. If we assume that all sows are supposed to be put into reproduction at the same age, then it is likely that these sows had certain problems preventing them to conceive earlier. If these problems had persisted, they could have been culled early based on the higher risk ratios. Similar results have been published by [2,16,17].

When the production level of the animals is included in the statistical model, the genetic value of the "functional" length of productive life can be approximated, as production is usually the main source of voluntary culling. Hopefully, selection on functional longevity would lead to a reduction in involuntary culling because of reproduction or health problems. When the production of the animal is not taken into account in the model, the genetic effect reflects the "true" longevity, which means that voluntary (i.e. for low production) and involuntary culling reasons are considered together.

In this paper, we have decided to focus on functional longevity, and therefore, we have included the number of piglets born alive relative to the annual herd's mean in our model, to account for phenotypic selection on litter size. Working only with absolute numbers of piglets would not be appropriate, because production at a young age is generally lower than at an age when the body is fully developed (milk production in cows [18], goats [19], litter size in sheep [20], litter size in pigs [21]). Also culling decisions based on litter size may vary with herd management and year. In other words, the same litter size can be treated differently in different farms or on the same farm in different years. It is therefore necessary to evaluate the farmers' decisions based on time and place, when and where they take place.

The risk ratios for the interaction between parity and RPC decreased within parity. This means that sows with a higher number of piglets born alive are clearly favoured, regardless of age of the sow or farrowing number. In most cases the risk of culling for sows with litters 25% above the herd's average is two to three times lower than for sows 25% under the herd's average. This was not the case at first parity, when the risk was only slightly lower for Large White sows, and without any clear tendency for nearly all classes of Landrace sows. The reason can be that the farmer did not want to cull the younger sows with a low number of piglets immediately, but rather wanted to give them another chance. Our results are similar to those of [4] for risk ratios between parities. Although in our case the parity number was included in an interaction term, the risk ratios followed a similar pattern.

The results can be compared better with the studies by [2,3] who also evaluated the risk of culling as an interaction term with parity number. In both cases, they found an increased risk of culling for sows with poor performance, which is similar to our results. There was a difference when comparing risk of culling between parities. Engblom et al. [2] have found that the risk of culling was relatively low for parities 2 to 7, while in our study, risks of culling were similar throughout the first parities and increased later in life. Brandt et al. [3] have used standardized values for the number of piglets born and have reported a relatively stable risk at the beginning of the productive life. They have concluded that culling decisions based on performance are made between parities 4 and 5. These results are in agreement with our findings, which show that the risk of culling was low for the first parities, and increased from parity 4 in Landrace sows. In Large White sows, the risk of culling was higher in parities 2 to 6, compared to the first parity, with an even more rapid increase from parity 7 onwards.

The risk of culling for effect of herd size change was similar for both breeds. The risk was highest for sows from herds that dramatically decreased in numbers with more than 50%. Decrease of farm size to such an extent could mean, that some extraordinary event happened (major financial problems or disease outbreak), which led to closure of the farm or extreme shrinkage of the herd. In the future, these records should be treated as right censored, because most likely the majority of the animals would live longer in normal circumstances.

In Large White herds for the second worst class, the risk was twice the one of the reference class, but it was stable in the case of only a slight decrease or increase in herd size. For the Landrace dataset the risk was extremely high for the worst class, but decreased rapidly in the second worst category, when the risk of culling was only 1.5 times higher for the Weibull model and 1.3 times higher for the grouped data model, in comparison with the reference class. Only a slight reduction in risk of culling for Large White herds increasing in size by more than 50% was detected. These results suggest that the farmer's decision to increase his herd size has a lower impact on keeping existing animals in the herd compared to the culling as a result of herd shrinkage. This indicates that the culling process on these farms continues in its usual way, probably because the expansion is done by introducing younger animals either from their own production or from other farms.

Growth performance undoubtedly influences the length of the productive life in pigs, and as such it should be included in the evaluation. New indexes for growth are calculated every two weeks in Austria and selection is based on these indexes. Including these in our models would be desirable, but unfortunately these indexes are not routinely saved. Nevertheless, the functional length of productive life could be modelled even better if they are added in the model.

### Genetic models

Most estimations of genetic variance in survival models have been based on sire or sire-maternal grand sire models [4,16,22,23]. Two reasons have justified such choices: first, the Laplace approximation of the posterior density of the genetic variance requires the repeated inversion of the Hessian matrix of the log-posterior density, which quickly becomes too time-consuming for large (animal model) applications. Due to the existence of time-dependent variables, this matrix is often less sparse than the usual mixed model coefficient matrix. Second, the quality of the Laplace approximation has been shown to depend on the number of observations per level of genetic effects [13]. Indeed, for many years, it was believed that animal models could not fit with the Survival Kit because of this alleged poor performance of the Laplace approximation. It has been shown recently (see [24] for references) that this concern was not justified when the data structure is adequate (several generations of related females). Increase in computing power has also made estimation easier for animal models on larger populations.

In this study, the sire model systematically led to the largest estimates of genetic variances (140 to 180% of the animal model genetic variance). However, it can be noted that there is a large uncertainty associated with the estimation of the genetic variance and that the overlap of its credible set with those of the other models is large. It has been claimed that the sire survival model is not consistent [25] because the error term of the survival models is not normally distributed and therefore does not properly include the remaining 3/4 of the genetic variance. One potential reason for the overestimation of the genetic variance may be a poor partition of the genetic variance between the sire variance and the error term.

In contrast, animal models gave low estimates of genetic variances and heritabilities. The other models gave estimates of genetic parameters very similar to the animal model in Landrace sows, but intermediate between the sire and animal models in Large White sows. The reason of this difference between breeds is unclear. Whatever the breed, the sire-mgs-dam within mgs does not present much advantage: the genetic variance estimates are almost exactly those obtained with the sire-mgs model and the dam component is small, not even representing the expected 3/16 of the genetic variance in Large White sows. The use of an animal model or a sire-mgs model to estimate the genetic variance seems advisable to account for all (or most) of the relationships in the population. The sire-mgs is less satisfactory from a modelling point of view but is favourable when the data structure is not adequate for the use of the animal model and also for easier computing.

Heritabilities in our study ranged between 0.08 and 0.14 for Large White and between 0.04 and 0.11 for Landrace sows depending on the model type. In general, heritabilities showed some variation, but after considering the standard error of estimates it seems that none of the genetic models is clearly superior to another.

To compare our results with those of other authors, we considered only the studies using survival analysis and not those using linear models, because estimates from these methods are not comparable [23].

Yazdi et al. [16] have published a rather wide range of heritabilities for longevity in Landrace populations ranging from 0.11 to 0.27. Serenius and Stalder [23] have found heritabilities around 0.16-0.17 for Landrace and 0.17-0.19 for Large White populations. It is important to note that, in both these papers, heritabilities were computed assuming that the residual effects followed an extreme value distribution with the variance π^2^/6, while we used the effective heritability according to [15]. This could potentially lead to differences in results depending on parameters of the Weibull distribution. For more details see [15]. In other words the direct comparison of heritabilities on log or original scale and the effective heritability is not possible.

To obtain more comparable results, we used the variances of [16,23] and inserted them in the equation of [15] (the same as we used for the sire model in our study). We used the sire variance of 0.037 in [16] using a model scenario similar to that in our study (herd*year treated as random, time dependent variable with 1 year intervals, with gamma parameter 5.76). Based on the estimated variance of the random herd*year effect and computed from the estimate of the gamma parameter, their effective heritability was 0.09. This result is very similar to our result of 0.07 from the sire model in the Landrace breed.

Using the same approach, we recalculated the heritability from [23] using the original sire's variances 0.068 to 0.081, resulting in effective heritabilities ranging between 0.18 and 0.21. These newly computed results were close to those in the paper (0.16-0.19) and were higher than our results in all cases. The herd*year effect was treated as a time-dependent fixed effect in this study, so no further adjustment was possible.

Engblom et al. [22] have used the same heritability definition as in our study, and found values of 0.06 for Landrace and 0.12 for Yorkshire (Large White) breeds with a sire survival model. Our study supports the previous findings that heritabilities for longevity in different breeds are not necessarily the same, even when using identical models. Their results were very similar to h^2 ^= 0.07 for Landrace and h^2 ^= 0.14 for Large White in our sire models.

### Continuous time vs. grouped data model

The production cycle (interval between two farrowings) in sows consists of three periods: gestation period, suckling period and days open (service period). The heritability for gestation length is 0.25-0.3 [26,27], but altering its length is not a breeding objective. In the Austrian Large White population, the mean gestation length is 114.8 ± 1.5 days, and in the Landrace 115.3 ± 1.5 days. The nursing period is heavily affected by breeders' decision for herd management. It is usually four weeks in Austria for both Large White (28.2 ± 5.0 days) and Landrace (28.2 ± 6.6 days) populations. The interval from weaning to first ovulation is optimally 5 to 6 days. If fertilisation is not successful, it can be longer with cycles repeated every 21 days. The time intervals for days open show much wider variation compared to gestation length and nursing period, with 16.0 ± 17.2 days in Large White and 14.0 ± 17.3 days in Landrace populations. From the average values and standard deviations of these three major components of the farrowing interval, it is obvious that the larger part of the variation is caused by days open. When considering days of productive life as the dependent variable, sows with longer reproductive cycles will tend to have higher estimated breeding values. This inconsistency is lifted when using grouped data models [6,8]. More reproductive cycles increase the breeding value of an animal not because it lives longer, but because it produces more.

## Conclusions

In this study, alternative models for the genetic evaluation of longevity in sows using continuous time and grouped data models in Large White and Landrace sows were examined. Risk of culling associated with length of productive life was influenced by all fixed and random effects. An increased risk was observed for animals having their first litter before 43 weeks or after 60 weeks of age, for sows from herds rapidly decreasing in size and sows with litter size under the herd's average in the given year. Some differences between the two breeds were observed, but in general the risks of culling for fixed effects in all models were comparable.

Heritabilities for the length of productive life were estimated using sire, sire-maternal grandsire, sire-dam, sire-maternal grandsire-dam within maternal grandsire and animal models with Weibull and grouped data models in both breeds. They ranged from 0.08 to 0.14 (s.e. 0.012-0.026) in Weibull and from 0.08 to 0.13 (s.e. 0.012-0.025) in grouped data models in Large White sows, and from 0.05 to 0.08 (s.e. 0.014-0.020) for the Weibull and from 0.07 to 0.11 (s.e. 0.016-0.023) for the grouped data models in Landrace sows. Sire-mgs models offer a good compromise between accurate modelling of the genetic part and computation requirements in case the data structure is not adequate to use an animal model. Heritabilities are low as for functional traits in general, but given the high economic importance of the length of productive life, it is really worthwhile to consider it as a selection criterion in pig breeding.

The grouped data models in pig tend to focus on the exact number of reproduction cycles rather than on the duration of productive life. Therefore sows with a higher number of parities are favoured, in contrast with sows which live longer just because of longer intervals between parities. The performance of grouped data models was comparable to continuous time models. Based on these results and because grouped data models reflect better the economical needs in meat animals, we conclude that grouped data models are more appropriate in pig.

The final outcome of the study is to provide basic information for routine genetic evaluation of the length of productive life in Austrian pig populations. The grouped data model will be used for its favourable contribution to deal with the non-productive days during a sow's lifetime.

## Competing interests

The authors declare that they have no competing interests.

## Authors' contributions

JS conceived the original idea of the study and reviewed the text. VD further developed the idea, helped with the analysis, wrote parts of the text and helped with the revision of existing parts. JP did the statistical analysis and helped to write the text. GM wrote the manuscript, did the initial data preparation and helped with the statistical analysis. All authors approved the final version.
